# Injurious Effect of Carbolic Acid from Injudicious Local Application

**Published:** 1873-01

**Authors:** John S. Hughson

**Affiliations:** Sumter, South Carolina


### INJURIOUS EFFECT OF CARBOLIC ACID FROM INJUDICIOUS LOCAL APPLICATION.

BY JOHN S. HUGHSON, OF SUMTER, SOUTH CAROLINA.

Never having seen any notice in regard to the injurious effect of carbolic acid when locally applied by the unprofessional, and consequently ignorant as regards the poisonous effects of this article, I transcribe the following from my note-book, as it may, perhaps, not be altogether void of interest:

August 27, 1872.—Called out this 9 o'clock P.M., to see a young gentleman who had noticed yesterday morning a slight discharge from the urethra, and having heard that carbolic acid was used in gonorrhœa, he concluded to test its value. Accordingly, he purchased one-half ounce of the saturated solution, and attempted to inject about one drachm within the urethra. The first few drops caused such intense pain, that he jerked the syringe from the urethra, and its contents were spilled upon the prepuce and under the skin of the penis.

When I saw it, some thirty minutes after, it was enormously swollen, and presented very much the appearance of phimosis.

The parts were totally insensible, there being no pain except within the urethral canal. Ordered cold water to be kept constantly applied, and directed—

℞.—Olive oil, f ʒ iv; laudanum, f ʒ ss. M. Sig.—Inject immediately, at midnight, and in the morning, using one-third at each injection.

28th.—Patient called at my office. The swelling of penis greatly reduced; can retract the prepuce entirely over the glans; the parts have a dull, whitish look; no pain. Continue the cold external application, and inject cold water frequently during the day.

29th.—Sent for this morning hurriedly. Arriving at the house, found the patient suffering intense pain from paraphimosis. He had retracted the prepuce for the purpose of washing the penis, and could not return it. Glans swollen and tender. Used the following:

℞.—Acet. plumbi, ℥ j; aquæ, ℥ viij. M.

Applied soft cloths saturated with the solution to the glans for ten or fifteen minutes; then attempted, and succeeded by manipulation, in restoring the parts to their natural position. The effects of the caustic were well marked by numerous raw surfaces on the inner part of the prepuce, and in streaks (as of a liquid running in a stream) upon the glans. Discharge during the night from the urethra, none this morning. Ordered cold application continued to the entire penis, and apply once every three hours to the raw surfaces with a camel's hair pencil, the following liniment, viz:

℞.—Linseed oil, lime water, aa ℥j. M.

Being in pain, gave fifteen drops of laudanum. Directed the patient to draw the prepuce back every two or three hours sufficiently to expose the corona glandis; I desired this retraction of the prepuce in order to guard against phimosis, which I still feared might take place.

30th.—Appearance of parts improved; but, it paining him to retract the prepuce as directed, he had neglected, or rather refrained from doing so. Phimosis strongly threatened. Omit the cold water, continue the liniment, and draw upon the skin *so as to expose the glans*, and thus prevent adhesions between the abraded surfaces in apposition, and consequent adherent phimosis. There is little gonorrhœal discharge; considerable pain in urinating. Prescribed as follows:

℞.—Carb. potass., ʒj; bro. potass., ʒij; aquæ ditil., ℥ij. S. Sig.—One teaspoonful three times a day.

31st.—Having neglected the direction to retract the prepuce, giving as a reason that it *pained* him, the grayish appearance of the skin having given away to raw surfaces, I find, as I feared, that there is a decided case of phimosis. Ordered a hot bath to be taken every three or four hours, and warm poultices constantly applied to the part. The pain, on micturition, is very severe. Ordered injection, just *before* urinating, of the linimentum calcis, with one-eighth grain of morphine in solution to be added.

Sept. 1st.—No prospect of removing the phimosis without an operation, but as the penis is still very sore from the effects of the carbolic acid, think best to await its further improvement; 'tis doing well, and looks much better; pain still severe on micturition, although the injection was of some benefit; directed now the injection to be used *immediately after* (instead of before) urinating, and inject cold water under the prepuce every hour or two. Urethral discharge has increased. Ordered—

℞.—Bals. copaiva, sweet spirits nitre, paregoric, aa f℥j; tinct. veratrum viride, fʒss. M. Sig.—One teaspoonful four times a day.

The parts continued to present an improved appearance, pain upon urinating considerably less, discharge from urethra almost entirely ceased, when, on the 6th of September, I operated. Having obtained from a mixture of chloroform, sulphuric ether and alcohol (recommended by a correspondent of your journal), complete anæsthesia, I clipped off, with the scissors, about one-fourth inch of the prepuce, extending over the glans; then, with the aid of the director and scalpel, slit up the prepuce near the frænum, thus permitting the glans to be entirely exposed to view. Upon recovering from the effects of chloroform, he was very nervous and pulse quite weak; gave brandy with one-eighth grain morphine, producing reaction and quiet; ordered cold-water dressing to the parts.

On the 8th, doing well, suppuration setting in; directed—

℞.—Carbolic acid crystals, ℥j; water, Oj. Sig.—One wine-glassful of this solution to be added to eight of cold water, and the part kept constantly wet with it.

Improved daily, and on the 1st of October was entirely well, with very little deformity of the organ, the glans being entirely covered by the prepuce; in fact, without the penis was raised up from its natural position, nothing abnornal would be at all noticed.

I have been surprised to find nothing said on the treatment, or even in regard to the injurious local effect of carbolic acid, when wrongfully used. Dr. Hill has an admirable treatise on "Carbolic Acid and its Uses," (American Journal of the Medical Sciences, July, 1872), and I have no doubt that many, with myself, would be glad to have the benefit of the experience of those who have had abundant opportunities in practicing upon its *abuses*.

I have thus referred particularly to the operation for phimosis, in order to direct the attention of those who are in the habit of dividing the prepuce *in front* to this method of operating—that is, close by the side of the frænum. 'Tis seldom that a ligature is required to arrest hemorrhage, as a suture may be passed on each side of the wound to connect the integument and mucous membrane, and thus obtain a more rapid recovery; there is very little apparent deformity following this operation.

Some eminent surgeons recommend this as the best for the patient, with but little more trouble to the operator.

NOTE.—Since the above was written, I see in the Reporter (September 29) an extract from the Bulletin de Therapeutique, citing cases of "dry gangrene following the use of carbolic acid." We learn that, though an article of excellent properties when judiciously employed, 'tis also a potent one for evil in the hands of the unskilled.—*Medical and Surgical Reporter.*

